# Extracorporeal Shockwave Therapy for Treating Chronic Low Back Pain: A Systematic Review and Meta-analysis of Randomized Controlled Trials

**DOI:** 10.1155/2021/5937250

**Published:** 2021-11-15

**Authors:** Lei Yue, Ming-shuai Sun, Hao Chen, Guan-zhang Mu, Hao-lin Sun

**Affiliations:** ^1^Department of Orthopaedics, Peking University First Hospital, Beijing 100034, China; ^2^Department of General Surgery, Peking University First Hospital, Beijing 100034, China; ^3^Department of Rehabilitation, Peking University First Hospital, Beijing 100034, China

## Abstract

**Objective:**

To assess the effectiveness and safety of extracorporeal shockwave therapy (ESWT) for the treatment of chronic low back pain (CLBP).

**Methods:**

This was a systematic review and meta-analysis of randomized controlled trials (RCTs) designed in accordance with the Preferred Reporting Items for Systematic Reviews and Meta-analysis statement standard. We identified relevant studies by searching multiple electronic databases, trial registries, and websites up to April 30, 2021, and examining reference lists. We selected RCTs that compared ESWT, in unimodal or multimodal therapeutic approaches, with sham ESWT or other active therapies. Two investigators independently extracted data and assessed the risk of bias and quality of the evidence. The main outcomes were pain intensity and disability status, examined as standardized mean differences (SMD) with 95% confidence intervals (CI). The risk of bias was assessed by using Cochrane Back and Neck (CBN) Group risk of bias tool and Jadad score, and GRADE was applied to determine the confidence in effect estimates. Heterogeneity was explored using sensitivity analysis and meta-regression.

**Results:**

Ten RCTs, including a total of 455 young to middle-aged individuals (29.2–55.8 years), were identified. Compared with control, the ESWT group showed lower pain intensity at month 1 (SMD = −0.81, 95% CI −1.21 to −0.42), as well as lower disability score at month 1 (SMD = −1.45, 95% CI −2.68 to −0.22) and at month 3 (SMD = −0.69, 95% CI −1.08 to −0.31). No serious shockwave-related adverse events were reported.

**Conclusion:**

The use of ESWT in CLBP patients results in significant and quantifiable reductions in pain and disability in the short term. However, further well-conducted RCTs are necessary for building high-quality evidence and promoting the application of ESWT in clinical practice.

## 1. Introduction

Low back pain refers to pain in the back area from the inferior costal margin to the gluteal fold. It affects individuals of all ages and is a markable contributor to diseases and healthcare burden worldwide [1]. The lifetime prevalence of low back pain could be as high as 84%, and the mean prevalence of low back pain has been estimated to be 11.9% [2, 3]. Most acute episodes of low back pain resolve within 6 weeks; however, about 25% of subjects with acute low back pain experience a recurrence in the following year, and chronic low back pain (CLBP) develops in up to 7% of the patients [4, 5]. CLBP is defined as low back pain that lasts longer than 12 weeks and commonly involves physical, behavioral, and socioeconomic factors [6, 7]. The aim of CLBP management is to reduce pain and improve quality of life while minimizing potential side effects. The basic noninvasive therapy of CLBP, recommended by existing clinical guidelines, includes bed rest, psychosocial therapy, pharmacotherapy, physical exercises, spinal manipulation, therapeutic ultrasound, and multidisciplinary rehabilitation programs [8–11]. Despite active treatment, only 31%–47% of CLBP patients fully recover within one year, indicating the necessity of more effective treatment methods for CLBP [12, 13].

Extracorporeal shockwave therapy (ESWT) is a noninvasive approach that passes shockwaves through the skin to the affected area. It was first introduced into clinical practice in 1982 for the management of urologic conditions [14]. However, owing to its noninvasive nature and negligible complications, the use of ESWT has been gaining in popularity worldwide for treating various musculoskeletal disorders, such as tendinitis, plantar fasciitis, humeral epicondylitis, and other soft tissue conditions, with a satisfying success rate of 65%–91% [15–17]. However, the current clinical guidelines do not recommend ESWT for CLBP management [8–11]. In a systematic review, Seco et al. concluded that there was not enough evidence to support the effectiveness of ESWT for treating low back pain (LBP) [18]. However, in the past decade, several randomized controlled trials (RCTs) were conducted on the topic of EWST for CLBP, yielding promising outcomes [19–24]; therefore, we think that an updated synthesis of the literature is needed.

The purpose of the systematic review described here was to evaluate the evidence on the effectiveness of ESWT for CLBP as reported in RCTs. A meta-analysis was also intended in case that there are subsets of data similar enough to be pooled.

## 2. Materials and Methods

The systematic review was developed in accordance with the Preferred Reporting Items for Systematic Review and Meta-Analysis (PRISMA) statement and the Method Guideline for Systematic Reviews in the Cochrane Back and Neck (CBN) Group [25, 26]. The protocol for this systematic review and meta-analysis was prospectively registered in the PROSPERO database (registration number: CRD42021250013).

### 2.1. Literature Search

RCTs that used ESWT in the treatment of CLBP in adults were selected in accordance with the recommendations of the CBN Group [26]. We searched the following electronic databases, registries, and websites on April 30, 2021, unrestricted by publication date or language:

1. *English Databases*. PubMed, Embase, Web of Science, SCOPUS, CINAHL (EBSCO), Cochrane Central Register of Controlled Trials (CENTRAL, via the Cochrane Library), and Physiotherapy Evidence Database (PEDro)
2. *Non-English Databases*. China National Knowledge Infrastructure (CNKI, China), Research Information Service System (RISS, South Korea), and J-Stage (Japan)
3. *Trial Registries*. World Health Organization (WHO) International Clinical Trials Registry Platform and ClinicalTrials.gov
4. *Websites*. Research Square and Google scholar

Grey literature (unpublished academic studies or thesis) was included.

The reference lists of retrieved trials and previous systematic reviews were also searched for citations of potentially eligible trials. The search strategy is shown in Supplementary File 1.

### 2.2. Selection Criteria

Inclusion criteria were as follows: (1) RCTs including adult patients (aged ≥ 18 years) who had experienced low back pain for over 3 months, regardless of age, gender, or ethnicity; (2) studies evaluating the effect of ESWT alone or in combination; and (3) control group received no treatment, sham procedures, pharmacotherapies, or other comparable interventions.

Exclusion criteria were as follows: (1) studies in which back pain of participants involved cervical spine, thoracic spine, coccyx, sacroiliac joint, or unidentifiable pain region; (2) studies that included participants with neurologic deficits and history of trauma, surgery, or inflammatory conditions such as ankylosing spondylitis; (3) non-RCT studies, such as cohort studies, case–control studies, case series, case reports, narrative reviews, editorials, and animal research; and (4) non-English studies in the absence of English abstract/figure/table captions or unsearchable by Google Scholar.

### 2.3. Study Selection and Extraction

During the first screening, two reviewers (H.C. and G.M.) evaluated the title and abstract of each citation and excluded irrelevant studies. For the second screening, two authors (L.Y. and M.S.) independently evaluated full-text articles using predetermined inclusion and exclusion criteria. In case of disagreement, a decision was made by team discussion with the corresponding author (H.S.).

Two independent investigators (L.Y. and M.S.) extracted the data on population characteristics, treatment intervention, control or comparators, and outcomes (PICO) using standardized forms (Supplementary File 2). The primary outcomes were as follows: (1) pain intensity, including Visual Analogue Scale (VAS), Numerical Rating Scale (NRS), and other scales (all pain scales were converted to a 10-point scale); and (2) back-specific disability score, i.e., Oswestry Disability Index (ODI). They were evaluated at two time points: at 1-month and 3-month follow-up. If data in a trial were available at multiple time points within each period, data at the nearest time point of each period were extracted. For example, study A recorded VAS score at 5-week follow-up and 6-week follow-up; the data 5 weeks would be extracted for “pain intensity at 1-month follow-up.” Adverse events were also recorded.

### 2.4. Dealing with Missing Data

When it was not possible to extract data of interest from a publication, the corresponding author was contacted via e-mail for unpublished data. The missing data was ignored if no response was received.

### 2.5. Risk of Bias (Quality) Assessment

The risk of bias for each of the included RCTs was assessed by using the bias tool recommended by the Cochrane Back and Neck (CBN) Group [26], and the graphical presentation of the risk of bias assessment was generated by RevMan 5.3 (Cochrane Collaboration, Software Update, Oxford, UK). The overall quality of each of the included trials was assessed by Jadad score [27]. Grading of Recommendations, Assessment, Development, and Evaluation (GRADE) approach was applied to evaluate the overall quality of the evidence based on the risk of bias, inconsistency, indirectness, imprecision, publication bias, and other factors. The GRADE approach evaluates the quality of evidence as *high*, *moderate*, *low*, or *very low* depending on the estimated effects [28]. Two independent reviewers (L.Y. and M.S.) performed the quality assessment, and conflicts were resolved through discussion.

### 2.6. Statistical Analysis

The results from the finally screened studies were combined to estimate the effective results as standardized mean differences (SMD) and 95% confidence intervals (CI) for continuous outcomes. The synthesis was done by generating a forest plot of the study estimates with R package meta, and random-effects model was used. Heterogeneity was examined by *I*^2^ and *τ*^2^ test [29]. Statistical significance was set at *p* < 0.05.

Only the outcomes of “pain intensity at 1-month follow-up” meet the definition of “large sample size (>400 patients)” by the Cochrane group, while the sample sizes of other outcomes were smaller than 400 [30]. Since the sensitivity analysis is usually not performed on small-sample-size outcomes, we only conducted sensitivity analysis on pain intensity at the 1-month follow-up. We attempted to explain heterogeneity for outcomes of sufficient sample size using meta-regression. Moreover, publication bias was examined by constructing funnel plots and performing Egger regression asymmetry test [31].

## 3. Results

### 3.1. Search Results and Description of the Studies

**Figure 1:**
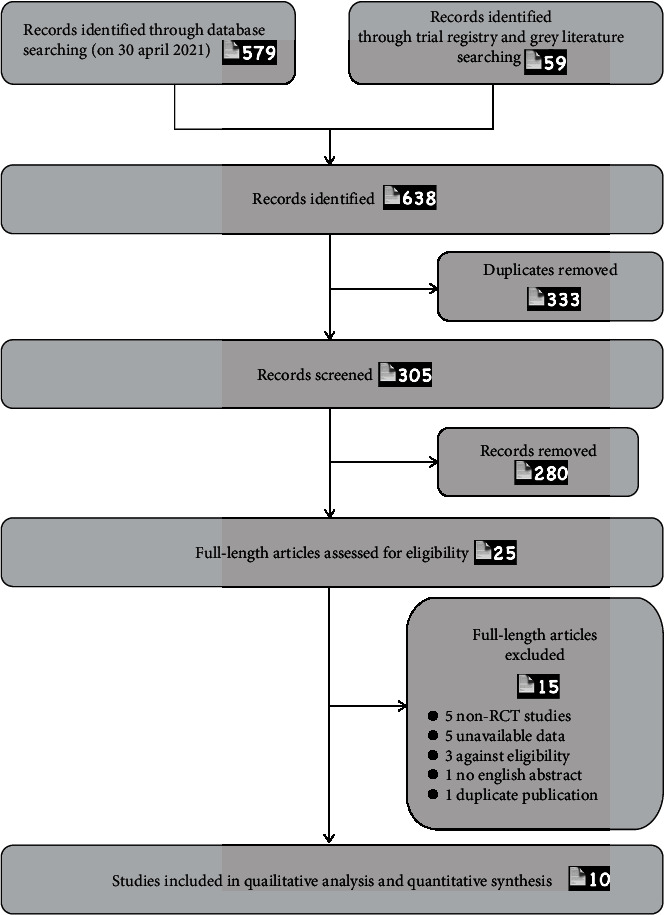
Flow diagram of the selection of studies.

The initial literature search yielded 638 studies; 333 duplicates were removed. After reviewing the titles and abstracts, 280 studies were excluded. Then, following a full-text review, we excluded five non-RCT articles, five RCTs with unavailable data, three studies not satisfying the eligible criteria, one study without English abstract/table, and one study of duplicate publication. Finally, 10 trials were included. The selection process is presented in a PRISMA flow diagram (Figure 1) [32].

### 3.2. Study Characteristics

The number of participants in these 10 studies was 455 in total and ranged from 30 to 91 participants, representing seven countries. The age of the participants ranged from 29.2 to 55.8 years. On average, there were more women than men (57.2% vs. 42.8%). The participants reported average pain at baseline from 4.2 to 8.4 out of 10.

The range of energy flux density (EFD) was 0.1–0.18 mJ/mm^2^. Treatment sessions varied from once per week for 3 weeks to twice per week over the course of 6 weeks. The cointerventions were sham ESWT or other active therapies (medication, physical exercise, transcutaneous electrical nerve stimulation (TENS), manipulation, exercise program, thermomagnetic therapy, and trigger point injection). These abovementioned elements are given in the “Summary of Findings” tables based on the PICO structure (Table 1) [33].

### 3.3. Methodological Quality

**Figure 2:**
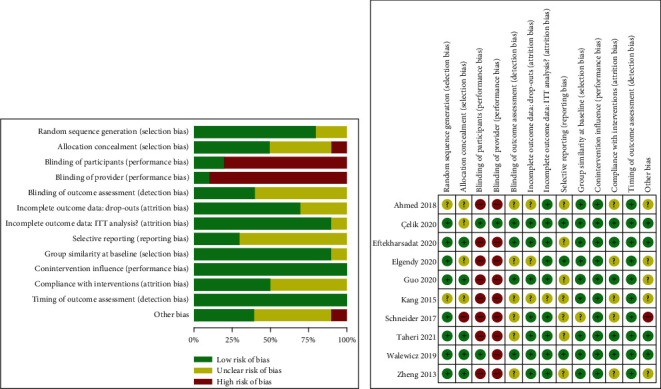
Risk of bias assessment of the included studies using the Cochrane Back and Neck Group (CBN) risk of bias tools.

The CBN Group risk of bias score for each study, with the key issued items being blinding, concealing, and compliance, is shown in Figure 2. Although the CBN Group does not recommend a cutoff for stratifying studies into those with high and low risk of bias [26], overall proportion of low risk of bias was 59.2%, so we consider that the included studies had a relatively low risk of bias (Figure 2). The mean Jadad score of the included studies was 3.4 (range, 1–5; Table 1), and eight out of 10 RCTs had a Jadad score ≥ 3 (indication of a methodologically good-quality trial [34]). Publication bias was assessed on the example of pain intensity at 1-month follow-up by visually inspecting funnel plots and Egger's tests; we did not detect any potential publication bias (Supplementary Figure B).

### 3.4. Reduction in Pain Intensity

**Figure 3:**
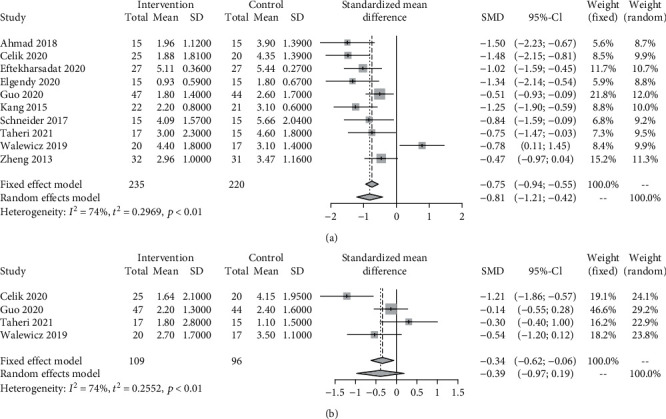
Standardized mean difference (SMD) with 95% confidence intervals (CI) of pain intensity at month 1 (a) and month 3 (b) for extracorporeal shock wave therapy (ESWT) versus comparators for chronic low back pain. Pooled SMD calculated by random-effects model.

Ten trials (455 patients) that reported pain intensity evaluation in 1 month from baseline and four trials (205 patients) that reported pain intensity evaluation in 3 months from baseline were included in the meta-analysis. The pooled results across all the studies showed that ESWT led to significantly greater reduction in pain intensity at month 1, compared with comparators (SMD = −0.81, 95% CI −1.21 to −0.42; *I*^2^ = 74%, *τ*^2^ = 0.2969). The pooled results at 3-month follow-up showed no significant reduction in pain intensity after ESWT compared with comparators (SMD = −0.39, 95% CI −0.97 to 0.19; *I*^2^ = 74%, *τ*^2^ = 0.26) (Figure 3). The GRADE score of pain intensity relief at 1-month follow-up was *low quality* and that at 3-month follow-up was *very low quality* (Supplementary Table C).

### 3.5. Improvement in Disability

**Figure 4:**
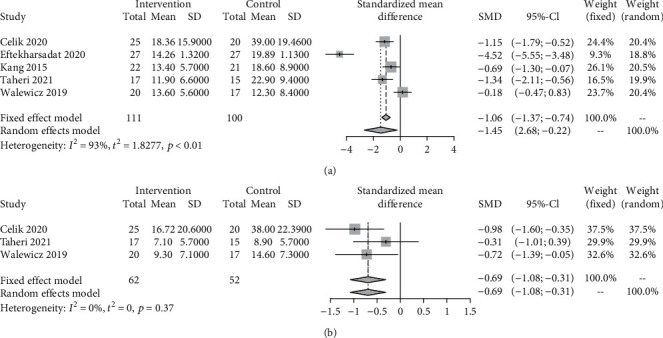
Standardized mean difference (SMD) with 95% confidence intervals (CI) of disability score at month 1 (a) and month 3 (b) for extracorporeal shock wave therapy (ESWT) versus comparators for chronic low back pain. Pooled SMD calculated by random-effects model.

Compared with other active comparators, ESWT trended toward more pronounced disability improvement at 1 month (5 trials, 211 patients: SMD = −1.45, 95% CI −2.68 to −0.22; *I*^2^ = 93%, *τ*^2^ = 1.83) and 3 months of follow-up (3 trials, 114 patients: SMD = −0.69, 95% CI −1.08 to −0.31; *I*^2^ = 0%, *τ*^2^ = 0) (Figure 4). The GRADE score of pain intensity relief at month 1 was *very low quality* and that at month 3 was *moderate quality* (Supplementary Table C).

### 3.6. Adverse Events

Half of the included studies examined adverse events, but most of these studies did not have unclear descriptions as to how and whether adverse events were registered systematically. Only the study by Kang mentioned that some patients experienced pain during ESWT procedure under the dose of 0.10–0.15 mJ/mm^2^ [35].

### 3.7. Sensitivity Analysis and Meta-regression

Sensitivity analysis was performed on the comparison of pain intensity at month 1. After excluding the unpublished trials (grey literature), the pooled SMD still showed that ESWT led to significantly larger reduction in pain intensity compared with comparators at 1-month follow-up, indicating robustness of the result (Supplementary Figure A).

We further conducted meta-regression for the comparison of pain intensity at 1-month follow-up between ESWT and comparator treatments. Three variables were included in the final model: age, female ratio, and baseline pain intensity score. However, none of these variables explained the statistical heterogeneity (Supplementary Table D).

## 4. Discussion

### 4.1. Statement of Principle Findings

Low-to-moderate-quality evidence showed that ESWT, either as a standalone or adjuvant approach for CLBP, was effective in relieving pain at 1-month follow-up and improving disability at 3-month follow-up compared with control. Although significantly lower disability score was also seen at 1-month follow-up between ESWT group and comparators, we considered these estimate effects as uncertain, owing to the very low quality of evidence, which calls for further RCTs to explore the effectiveness of ESWT. Additionally, there were no notable ESWT-related adverse events, except for one trial which reported treatment-associated pain.

### 4.2. Comparison with Other Studies

To our knowledge, this review is the first to have conducted a meta-analysis of RCTs on the effectiveness of ESWT on CLBP. The effect of ESWT on low back pain has previously been reviewed by several studies, but none of them were qualified enough to provide qualified evidence. An unpublished systematic review and meta-analysis of five RCTs on the effect of ESWT in treating low back pain indicated that the pooled mean difference in pain intensity and disability score were lower in the ESWT group than those in the control group [36]. However, the overall sample size was small (222 participants in total), which may have led to serious imprecision. Moreover, the GRADE scale was not used for evaluating the estimated effects; the study was not registered or published, and the time points for post-treatment evaluation were not set. In addition, obvious errors were found in the manuscript; a protocol for systematic review and meta-analysis of ESWT on low back pain was published by Wei et al., but no subsequent work by the team was found [37]; a systematic review by Seco et al. investigated the effectiveness of shockwave therapy for low back pain, but they only included one trial of *Fairmed* device, which was actually not a shockwave device, as noted by Ramon et al. [18, 38]; the most recent systematic review of ESWT on CLBP by Walewicz included six trials; however, the finally included studies were in contradiction with the eligibility criteria of the study, and the data were not pooled for estimation [39].

### 4.3. Implications for Clinicians and Researchers

In most evidence-based clinical guidelines, ESWT has still not been recommended or presented as a therapeutic option for CLBP due to the lack of sufficient evidence [40–44]. The only exception is the expert consensus by Chinese Association for the Study of Pain (CASP), in which ESWT has been listed as an alternative for treating back pain due to disc herniation [45]; however, the strength of this recommendation is questionable due to very low quality evidence [19, 37, 46].

Though still neglected by guidelines, the effectiveness of using ESWT for treating CLBP was proven by several RCTs in the past decade. The included studies showed that ESWT, delivered as a standalone therapy or in combination with other active therapies, resulted in clinical outcomes superior to those achieved with the guideline-recommended approaches. When analyzing the pre- and post-treatment differences, ESWT in most trials achieved a minimal clinically important change (MCIC) (change in VAS, NRS, and ODI over 2, 2.5, and 10, respectively) at the 1-month and 3-month follow-ups in terms of pain relief as well as disability improvement [47]. Regarding heterogeneity analysis, subgroup analysis was not performed here because the insufficient number of study participants could result in an inability to show differences [48], and no significant variables explained the statistical heterogeneity for the outcome by meta-regression. Additionally, no appreciable difference in terms of the analgesic effect of ESWT versus comparators at 1-month follow-up was found after excluding unpublished trials, indicating high stability of the analysis. Further well-constructed studies are needed to identify best possible treatment strategies for specific subgroups.

As for the safety concerns, it is difficult to assess the incidence of adverse events based on the included studies due to unclear descriptions. According to the guidelines of the International Society for Medical Shockwave Treatment (ISMST), for myofascial syndrome treatment, some adverse events, such as transient increase in pain and very rarely hematoma, could occur [49]. Thus, patients should be fully informed of potential risks prior to ESWT treatment.

### 4.4. Strengths and Limitations

The main strengths of this review include the following: (1) the use of a prespecified protocol registered on PROSPERO; (2) the use of systematic and explicit search strategy and eligibility criteria to include all of the eligible trials, including grey literature and non-English studies, which may reduce publication bias; and (3) the use of the CBN Group risk of bias tool and Jadad score to assess methodological quality of the included trials and the GRADE system to determine the overall quality of each critical outcome. None of the authors of the present study reported any conflicts of interests.

The limitations of this review are as follows: (1) the outcomes were based on trials with a small sample size, which might have overestimated the effect size and hindered planned subgroup analysis; (2) the trials included were clinically diverse in etiology, duration of pain, sessions/dose/timing of treatment, and comparators, causing heterogeneity in effect estimates and limited generalizability of the evidence; and (3) the long-term follow-up and data from ongoing trials were not available. With these limitations, the results should be interpreted with caution. Future trials with large sample size are anticipated to replicate our results. In addition, future studies should be more specific about randomization and conceal allocation, use blinding of patients and assessors, and design well-defined subgroups to establish optimal treatment strategy for different populations.

## 5. Conclusions

Based on the current state of the literature, the use of ESWT in CLBP patients results in significant and quantifiable reductions in pain at 1-month follow-up and disability at 3-month follow-up. However, ESWT should be implemented with caution, and further well-conducted RCTs are necessary to build qualified evidence and promote the application of ESWT in clinical practice.

## Figures and Tables

**Table 1:** Summary of findings of the systematic review of extracorporeal shock wave therapy (ESWT) for chronic low back pain (CLBP).

	Characteristics of the included studies (based on PICO structure) [33]								
	Patients		Intervention	Comparison	Outcomes (ESWT vs. control)				Quality of evidence
Author	ESWT vs. control: randomized (completed); mean age (yrs); gender	Eligibility criteria	ESWT group: device; parameters; adjuvant therapies	Control group: comparator therapies	Pain results	Disability/functional/quality of life results	Adverse events	Author's conclusions	Jaded score
Ahmed et al. 2018 [24]	*N*: 15 (15) vs. 15 (15) Age: 29.40 ± 2.67 vs. 29.20 ± 3.05 Gender (female): 15/15 vs. 15/15 BMI: 26.68 ± 1.77 vs. 25.81 ± 1.97	Inclusion: multiparous women suffering from postpartum low back pain (3 mo after delivery); age, 25–35 yrs; BMI < 30 kg/m^2^; parity, 2–3 children; delivery type, normal cesarean section Exclusion: gynecological diseases; specific spinal diseases	ESWT Device: Storz with 20 mm D-actor head Parameters: 2 bar, 10 Hz, 2000 pulses, 0.18 mJ/mm^2^, 5 min Sessions: twice a wk for 4 wk Physical exercise: abdominal strengthening exercise, postural correction exercises, and posterior pelvic tilting exercise (60 min) Sessions: twice a wk for 4 wk	Physical exercise: abdominal strengthening exercise, postural correction exercises, and posterior pelvic tilting exercise (60 min) Sessions: twice a wk for 4 wk	VAS Baseline: 8.07 ± 1.25 vs. 8.73 ± 1.18 4-wk FU: 1.96 ± 1.12 vs. 3.90 ± 1.39		ND	Shockwave therapy is an effective modality in alleviating postpartum low back pain	1

Çelik et al. 2020 [50]	*N*: 25 (25) vs. 25 (20) Age: 40.76 ± 10.68 vs. 40.25 ± 10.26 Gender (female): 10/25 vs. 12/20	Inclusion: 18 and 65 yrs old; CLBP history ≥ 3 months; history of physical therapy and/or spinal injection within the past 3 mo Exclusion: Specific spinal disease; pregnancy; cardiac pacemaker; rheumatic diseases; structural anomaly; medical treatment such as pregabalin, gabapentin, and antidepressant for chronic pain treatment	ESWT Device: EMD, E1000, C-ARMOR, 2011, Turkey Parameters: 2.5 Hz, 1500 shocks, 0.12 mJ/mm^2^, 20 min, twice a wk for 6 wk	Sham ESWT Device: EMD, E1000, C-ARMOR, 2011, Turkey Parameters: 2.5 Hz, 1500 shocks, 0.08 mJ/mm^2^, 20 min, twice a wk for 6 wk	NRS Baseline at rest: 5 (2–8) vs. 5 (3–8) Baseline at movement: 8 (4–10) vs. 8.5 (7–10) 6-wk FU at rest: 1.88 ± 1.81 vs. 4.35 ± 1.39 6-wk FU at movement: 3.88 ± 2.42 vs. 7.25 ± 1.65 12-wk FU at rest: 1.64 ± 2.10 vs. 4.15 ± 1.95 12-wk FU at movement: 3.16 ± 3.00 vs. 7.05 ± 2.42	ODI Baseline: 44 (11–74) vs. 46 (15–82) 6-wk FU: 18.36 ± 15.90 vs. 39.00 ± 19.46 12-wk FU: 16.72 ± 20.60 vs. 38.00 ± 22.39	None of the patients left the study due to side effects of the treatment	ESWT showed a significant superiority over placebo in improving the parameters of pain, disability, depression, anxiety, and quality of life in the patients with CLBP	5

Eftekharsadat et al. 2020 [23]	*N*: 27 (27) vs. 27 (27) Age: 44.74 ± 9.34 vs. 45.04 ± 11.86 Gender (female): 20/27 vs. 17/27 BMI: 27.47 ± 1.44 vs. 26.20 ± 2.06	Inclusion: CLBP history ≥ 3 months; quadratus lumborum muscle tenderness; palpable nodule/tout band; normal neurological examination; VAS > 4/10 Exclusion: treatment in past 3 mo; SIJ problems; Bertolotti syndrome; hemorrhagic disorders; infection; allergy to corticosteroids; pregnancy; diabetes; dynamic listhesis; BMI > 30 kg/m^2^	ESWT Device: Zimmer enPulsPro Medizin system GmbH, Germany Parameters: 10–16 Hz, 1500 pulses, 0.1 mJ/mm^2^ Sessions: once a wk for 3 wk Physical exercise: stretching exercises	TPI Muscle injection of 40 mg triamcinolone +2 mL lidocaine 2%; one session Physical exercise: stretching exercises	VAS Baseline: 7.63 ± 0.27 vs. 7.22 ± 0.26 2-wk FU: 5.81 ± 0.25 vs. 3.52 ± 0.35 4-wk FU: 5.11 ± 0.36 vs. 5.44 ± 0.27	ODI Baseline: 22.44 ± 1.44 vs. 23.93 ± 1.27 2-wk FU: 16.07 ± 1.29 vs. 13.52 ± 1.13 4-wk FU: 14.26 ± 1.32 vs. 19.89 ± 1.13 SF-36 Baseline: 74.59 ± 1.35 vs. 73.78 ± 1.30 2-wk FU: 81.93 ± 1.53 vs. 79.56 ± 1.46 4-wk FU: 84.00 ± 1.48 vs. 75.48 ± 1.08	No clinically important adverse events, side effects, or severe complications requiring medical interference were mentioned in either of the groups	After 4 wk of treatment, ESWT was more efficacious than corticosteroid TPI in reducing pain and improving quality of life and disability outcomes	4

Elgendy et al. 2020 [51]	*N*: 15 (15) vs. 15 (15) Age: 32.73 ± 6.73 vs. 33.26 ± 5.48 Gender (female): 5/15 vs. 5/15 BMI: 24.93 ± 1.72 vs. 25.56 ± 1.27	Inclusion: 20–30 yrs old; normal BMI; CLBP history > 3 mo Exclusion: specific spinal disease; pregnancy	ESWT Device: Elettronica Paganis medical devices with 17 mm head Parameters: 5 Hz, 2000 pulses, 0.10 mJ/mm^2^ Sessions: twice a wk for 6 wk Physical therapy: manual passive stretching exercises, progressive strengthening exercises for abdominal and back muscles, anterior and posterior pelvic tilt; physical therapy program was applied twice per wk for 6 wk	Physical therapy: manual passive stretching exercises, progressive strengthening exercises for abdominal and back muscles, anterior and posterior pelvic tilt; physical therapy program was applied twice per wk for 6 wk	VAS: Baseline: 7.46 ± 1.88 vs. 7.2 ± 2.04 6-wk FU: 0.93 ± 0.59 vs. 1.8 ± 0.67		ND	ESWT can effectively improve trunk muscle activity and reduce pain level in patients with nonspecific CLBP	3

Guo et al. 2020 [52]	*N*: 47 (47) vs. 48 (44) Age: 34.9 ± 8.7 vs. 36.0 ± 11.2 Gender (female): 22/47 vs. 25/48 BMI: 22.3 ± 3.0 vs. 22.7 ± 3.2	Inclusion: 18–80 yrs old; CLBP history > 3 mo Exclusion: specific spinal disease, history of spine surgery, mental illness, uncontrolled systemic diseases	ESWT Device: Swiss DolorClast device (Electro Medical Systems, Nyon, Switzerland) and EvoBlue handpiece Parameters: 15 Hz, 4000 pulses Sessions: once a wk for 4 wk	Medication Celecoxib (1 × 200 mg per day for moderate pain (NRS score 4–6), or 2 × 200 mg per day (NRS score 7–10)) and eperisone (3 × 50 mg per day) for 4 wk	NRS Baseline: 4.2 ± 1.2 vs. 4.2 ± 1.5 4-wk FU: 1.8 ± 1.4 vs. 2.6 ± 1.7 12-wk FU: 2.2 ± 1.3 vs. 2.4 ± 1.6	PSEQ Baseline: 4.6 ± 4.1 vs. 6.9 ± 4.7 4-wk FU: 2.9 ± 2.9 vs. 4.8 ± 5.4	No severe adverse events were observed during the study	rESWT may be superior to medication in reducing pain in subjects with CLBP	5

Kang 2015 [35]	*N*: 22 vs. 21 Age: 43.1 BMI: 21.33	Inclusion: CLBP history > 6 mo; diagnosis of myofascial pain syndrome Exclusion: specific spinal disease, history of spine surgery, mental illness	ESWT Device: Evotron, Switech Medical, Switzerland Parameters: 4 Hz, 1000 pulses, 0.10–0.15 mJ/mm^2^ Sessions: once a wk for 8 wk Conservative treatment: resting, medication, heat therapy, TENS, and therapeutic exercise Exercise was applied 3/wk for 8 wk	Conservative treatment: resting, medication, heat therapy, TENS, and therapeutic exercise Exercise was applied 3/wk for 8 wk	VAS: Baseline: 5.4 ± 1.6 vs. 5.3 ± 1.8 8-wk FU: 2.2 ± 0.8 vs. 3.1 ± 0.6	ODI: Baseline: 28.4 ± 11.2 vs. 28.9 ± 10.3 8-wk FU: 13.4 ± 5.7 vs. 18.6 ± 8.9 SF-36 Baseline: 36.7 ± 7.8 vs. 37.2 ± 8.2 8-wk FU: 52.3 ± 8.5 vs. 45.6 ± 8.4	Pain during ESWT was observed in some patients	ESWT together with the conservative rehabilitation therapy has a great influence on the myofascial pain syndrome	1

Schneider et al. 2017 [20]	*N*: 15 (15) vs. 15 (15) Age: 43.2 (range: 23–65) Gender (female): 18/30	Inclusion: CLBP history ≥ 3 mo; legal age Exclusion: major disease; drug addiction; mental illness; pregnancy	ESWT Device: Cellconnect Impulse Parameters: 15–42 Hz Sessions: twice a wk for 3 wk Myofascial trigger therapy: palpation of the target musculature, identification of the trigger points, and provocation of the taut muscle fasciae for 30 min Sessions: twice a wk for 3 wk	Myofascial trigger therapy: palpation of the target musculature, identification of the trigger points, and provocation of the taut muscle fasciae for 30 min Sessions: twice a wk for 3 wk	7-point-Likert scale: ESWT + MT vs. MT Baseline: 4.8 ± 0.9 vs. 4.2 ± 1.0 3-wk FU: 2.6 ± 1.0 vs. 3.6 ± 1.3		All 30 patients completed the trial, and none complained about adverse effects	Combining MT with ESWT enhances the physiotherapeutic effectiveness of treating chronic back pain	3

Taheri et al. 2021 [53]	*N*: 19 (17) vs. 19 (15) Age: 42.5 ± 10.1 vs. 37.1 ± 11.8 Gender (female): 11/17 vs. 6/15 BMI: 27.1 ± 5.5 vs. 26.8 ± 2.1	Inclusion: age > 18 yrs; CLBP history > 3 mo Exclusion: undergoing treatment or surgery; pregnancy; cognitive problems; specific spinal diseases; medical condition; uncontrolled systemic diseases	ESWT Device: DUOLITH SD1, Storz Medical, Tägerwilen, Switzerland Parameters: 4 Hz, 1500 pulses, 0.15 mJ/mm^2^ Sessions: once a wk for 4 w Oral medications and exercise program: oral medications (tizanidine hydrochloride and meloxicam); exercise program: stretching exercises	Sham ESWT: method of sham treatment: treatment with same sound but no energy Oral medications and exercise program: oral medications (tizanidine hydrochloride and meloxicam). Exercise program: stretching exercises	VAS ESWT vs. control Baseline: 6.6 ± 1.8 vs. 6.8 ± 1.9 4-wk FU: 3.0 ± 2.3 vs. 4.6 ± 1.8 12-wk FU: 1.8 ± 2.8 vs. 1.1 ± 1.5	ODI ESWT vs. control Baseline: 41.1 ± 21.2 vs. 40.5 ± 19.1 4-wk FU: 11.9 ± 6.6 vs. 22.9 ± 9.4 12-wk FU: 7.1 ± 5.7 vs. 8.9 ± 5.7	ND	ESWT, along with oral medication and exercise therapy, appears to be a safe and effective method in the short-term treatment of CLBP patients	4

Walewicz et al. 2019 [19]	*N*: 20 (20) vs. 20 (17) Age: 51.1 ± 8.4 vs. 55.8 ± 9.3 Gender (female): 14/20 vs. 15/20 BMI: 29.0	Inclusion: CLBP of L5-S1 discopathy; CLBP history > 3 mo Exclusion: acute spinal pain; discopathy on a different level of the spine; lack of pain and reduced mobility in the lumbar and sacral regions; specific spinal diseases; pregnancy; pacemaker; cardiovascular diseases; blood coagulation disorders; metal implants; mental disorders; cancer; psoriasis; scleroderma; viral and bacterial infections; history of spinal surgery or drug therapy	ESWT Device: Cosmogamma, Indonesia Parameters: 2.5 bars, 5 Hz, 2000 pulses, 0.1 mJ/mm^2^, 7 min Sessions: twice a wk for 5 wk Stability training: physical improvement in the form of functional training (45 min, once a day, 5 days a wk)	Sham ESWT: special polyethylene applicator cap with the same sound signals during the procedure of the pneumatic head and the same technical parameters as in the real procedures Stability training: physical improvement in the form of functional training (45 mins, once a day, 5 days a wk)	VAS Baseline: 4.7 ± 1.9 vs. 4.7 ± 1.4 5-wk FU: 4.4 ± 1.8 vs. 3.1 ± 1.4 9-wk FU: 2.7 ± 1.7 vs. 3.5 ± 1.1 17-wk FU: 2.0 ± 2.0 vs. 4.4 ± 1.2 LPS Baseline: 6.3 ± 2.0 vs. 6.2 ± 2.8 5-wk FU: 5.7 ± 2.4 vs. 4.3 ± 2.1 9-wk FU: 3.9 ± 1.8 vs. 5.2 ± 2.2 17-wk FU: 2.2 ± 2 vs. 6.4 ± 2.6	ODI Baseline: 16.1 ± 5.2 vs. 16.1 ± 8.0 5-wk FU: 13.6 ± 5.6 vs. 12.3 ± 8.4 9-wk FU: 9.3 ± 7.1 vs. 14.6 ± 7.3 17-wk FU: 9.3 ± 8.7 vs. 17.8 ± 7.2	ND	ESWT had a significant effect on the reduction of pain and the improvement of functional condition compared with the conventional physiotherapy program in patients with LBP	5

Zheng et al. 2013 [22]	*N*: 33 (32) vs. 33 (31) Age: 45.84 ± 11.85 vs. 47.39 ± 12.69 Gender (female): 14/32 vs. 6/31	Inclusion: age 18–60 yrs; LBP >12 wk without treatment Exclusion: specific spinal diseases; serious systemic diseases	ESWT Device: SHOCKMASTER-500, Gymna, Belgium Parameters: 1.6–3.0 bar, 8–12 Hz, 2000 pulses Sessions: twice a wk for 2 wk	Thermomagnetic therapy Sessions: 40°C, 15 min Sessions: once a day for 14 days	VAS: Baseline: 6.32 ± 1.12 vs. 6.24 ± 1.19 2-wk FU: 2.96 ± 1.00 vs. 3.47 ± 1.16	FFD: Baseline: 25.72 ± 9.63 vs. 26.42 ± 9.83 2-wk FU: 12.00 ± 4.89 vs. 16.94 ± 6.83	ND	The pneumatically ballistic extracorporeal shockwave is more effective for chronic nonspecific low back pain than hot magnet	3

ESWT: extracorporeal shockwave therapy; FFD: finger foot distance; FU: follow-up; LPS: Laitinen Pain Scale; ND: not described; NRS: Numeric Rating Scale; ODI: Oswestry Disability Index; PLC: Profil der Lebensqualität chronisch Kranker; SIJ: sacroiliac joint; TPI: trigger point injection; VAS: Visual Analogue Scale; TENS: transcutaneous electrical nerve stimulation; PSEQ: Pain Self-Efficacy Questionnaire. Specific spinal diseases include discopathy with or without radiculopathy/cauda equina syndrome, spondylosis, spondylolisthesis, spinal malignancies, spinal fractures, spinal infections, and spinal trauma. One month equaled 4 weeks in this research.

## Data Availability

The sources of data used in this study are available within the manuscript and its supplementary files. Other data are available from the corresponding author upon reasonable request.

## Supplementary Materials

Supplementary 1 Supplementary file 1: search protocol, sensitivity test, publication bias test, subgroup analysis, and meta-regression. Table A: search strategy and results of literature search. Table B: quality assessment of included studies by CBN Group risk of bias tool and Jadad score. Table C: quality of evidence assessment by GRADE. Table D: table of meta-regression results of the variables of pain intensity of 1-month follow-up. Figure A: sensitivity analysis: forest plots of pain intensity at 1-month follow-up after removing unpublished trials. Figure B: publication bias analysis: Egger's test and funnel plots of pain intensity at 1-month follow-up.

Supplementary 2 Supplementary file 2: data extraction form (*Supplementary materials*).

## References

[1] Airaksinen O., Brox J. I., Cedraschi C. (2006). Chapter 4. European guidelines for the management of chronic nonspecific low back pain. *European Spine Journal*.

[2] Hoy D., Bain C., Williams G. (2012). A systematic review of the global prevalence of low back pain. *Arthritis and Rheumatism*.

[3] Walker B. F. (2000). The prevalence of low back pain: a systematic review of the literature from 1966 to 1998. *Journal of Spinal Disorders*.

[4] Speed C. (2004). Low back pain,. *BMJ*.

[5] Stanton T. R., Henschke N., Maher C. G., Refshauge K. M., Latimer J., McAuley J. H. (2008). After an episode of acute low back pain, recurrence is unpredictable and not as common as previously thought. *Spine*.

[6] Rubinstein S. M., van Middelkoop M., Assendelft W. J., de Boer M. R., van Tulder M. W., Cochrane Back and Neck Group (2011). Spinal manipulative therapy for chronic low-back pain. *Cochrane Database of Systematic Reviews*.

[7] Morlion B. (2013). Chronic low back pain: pharmacological, interventional and surgical strategies. *Nature Reviews. Neurology*.

[8] National Guideline Centre (UK) (2016). *Low Back Pain and Sciatica in Over 16s: Assessment and Management*.

[9] Scott Kreiner D. (2020). Evidence-based clinical guidelines for multidisciplinary spine care: diagnosis & treatment of low back pain. *North American Spine Society*.

[10] Oliveira C. B., Maher C. G., Pinto R. Z. (2018). Clinical practice guidelines for the management of non-specific low back pain in primary care: an updated overview. *European Spine Journal*.

[11] Qaseem A., Wilt T. J., McLean R. M., Forciea M. A., for the Clinical Guidelines Committee of the American College of Physicians (2017). Noninvasive treatments for acute, subacute, and chronic low back pain: a clinical practice guideline from the American College of Physicians. *Annals of Internal Medicine*.

[12] Costa L. . C. M., Maher C. G., McAuley J. H. (2009). Prognosis for patients with chronic low back pain: inception cohort study. *BMJ*.

[13] da Silva T., Mills K., Brown B. T. (2019). Recurrence of low back pain is common: a prospective inception cohort study. *Journal of Physiotherapy*.

[14] Chaussy C., Schmiedt E., Jocham B., Brendel W., Forssmann B., Walther V. (1982). First clinical experience with extracorporeally induced destruction of kidney stones by shock waves. *The Journal of Urology*.

[15] Speed C. A. (2004). Extracorporeal shock-wave therapy in the management of chronic soft-tissue conditions. *Journal of Bone and Joint Surgery. British Volume (London)*.

[16] Wang C. J. (2012). Extracorporeal shockwave therapy in musculoskeletal disorders. *Journal of Orthopaedic Surgery and Research*.

[17] Ogden J. A., Alvarez R. G., Levitt R., Marlow M. (2001). Shock wave therapy (Orthotripsy) in musculoskeletal disorders. *Clinical Orthopaedics and Related Research*.

[18] Seco J., Kovacs F. M., Urrutia G. (2011). The efficacy, safety, effectiveness, and cost-effectiveness of ultrasound and shock wave therapies for low back pain: a systematic review. *The Spine Journal*.

[19] Walewicz K., Taradaj J., Rajfur K. (2019). The effectiveness of radial extracorporeal shock wave therapy in patients with chronic low back pain: a prospective, randomized, single-blinded pilot Study. *Clinical Interventions in Aging*.

[20] Schneider R. (2018). Effectiveness of myofascial trigger point therapy in chronic back pain patients is considerably increased when combined with a new, integrated, low-frequency shock wave vibrotherapy (Cellconnect Impulse): a two-armed, measurement repeated, randomized, controlled pragmatic trial. *Journal of Back and Musculoskeletal Rehabilitation*.

[21] Sukhon T. (2018). Effects of radial shockwave therapy for reducing lower back pain caused by chronic muscle strain. *Science and Technology Asia*.

[22] Zheng Z., Gao Q., Wang J. (2013). Effect of pneumatically ballistic extracorporeal shockwave on chronic nonspecific low back pain. *Chinese Journal of Rehabilitation Theory and Practice*.

[23] Eftekharsadat B., Fasaie N., Golalizadeh D. (2020). Comparison of efficacy of corticosteroid injection versus extracorporeal shock wave therapy on inferior trigger points in the quadratus lumborum muscle: a randomized clinical trial. *BMC Musculoskeletal Disorders*.

[24] Nahas E. M., Ahmed D. S., Magda S. M., Fayiz F. (2018). Effect of shock wave therapy on postpartum low back pain. *The Medical Journal of Cairo University*.

[25] Shamseer L., Moher D., Clarke M. (2015). Preferred reporting items for systematic review and meta-analysis protocols (PRISMA-P) 2015: elaboration and explanation. *BMJ*.

[26] Furlan A. D., Malmivaara A., Chou R. (2015). 2015 updated method guideline for systematic reviews in the Cochrane Back and Neck Group. *Spine*.

[27] Jadad A. R., Moore R. A., Carroll D. (1996). Assessing the quality of reports of randomized clinical trials: is blinding necessary?. *Controlled Clinical Trials*.

[28] Guyatt G., Oxman A. D., Akl E. A. (2011). GRADE guidelines: 1. Introduction--GRADE evidence profiles and summary of findings tables. *Journal of Clinical Epidemiology*.

[29] Rücker G., Schwarzer G., Carpenter J. R., Schumacher M. (2008). Undue reliance on I 2 in assessing heterogeneity may mislead. *BMC Medical Research Methodology*.

[30] Ryan R., Hill S. (2016). *How to GRADE the Quality of the Evidence*.

[31] Egger M., Smith G. D., Schneider M., Minder C. (1997). Bias in meta-analysis detected by a simple, graphical test. *BMJ*.

[32] Page M. J., McKenzie J. E., Bossuyt P. M. (2021). The PRISMA 2020 statement: an updated guideline for reporting systematic reviews. *BMJ*.

[33] Richardson W. S., Wilson M. C., Nishikawa J., Hayward R. S. (1995). The well-built clinical question: a key to evidence-based decisions. *ACP Journal Club*.

[34] Kjaergard L. L., Villumsen J., Gluud C. (2001). Reported methodologic quality and discrepancies between large and small randomized trials in meta-analyses. *Annals of Internal Medicine*.

[35] Kang J. (2015). *The Effect of Extracorporeal Shock Wave Therapy on Chronic Low Back Pain Patients due to Myofascial Pain Syndrome*.

[36] Ma J., Yan Y., Wang B., Sun W., Yue D., Wang W. (2020). Effectiveness and safety of extracorporeal shock wave treatment for low back pain: a systematic review and meta-analysis of RCTs.

[37] Wei W., Tang H.-y., Li Y.-z., Wang T.-s. (2019). Effectiveness of extracorporeal shock wave for low back pain. *Medicine*.

[38] Ramon S., Leal C., Schaden W., Moya D., Guiloff L., Freitag K. (2015). Improving methodology when analyzing shockwave evidence. *The Spine Journal*.

[39] Walewicz K. (2020). Extracorporeal shock wave therapy (ESWT) in chronic low back pain: a systematic review of randomized clinical trials. *Medical Science Pulse*.

[40] Low back pain and sciatica in over 16s: assessment and management. https://www.nice.org.uk/guidance/ng59.

[41] Arnau J. M., Vallano A., Lopez A., Pellisé F., Delgado M. J., Prat N. (2006). A critical review of guidelines for low back pain treatment. *European Spine Journal*.

[42] Bailly F., Trouvin A. P., Bercier S. (2021). Clinical guidelines and care pathway for management of low back pain with or without radicular pain. *Joint, Bone, Spine*.

[43] Ma K., Zhuang Z. G., Wang L. (2019). The Chinese Association for the Study of Pain (CASP): consensus on the assessment and management of chronic nonspecific low back pain. *Pain Research & Management*.

[44] Pillastrini P., Gardenghi I., Bonetti F. (2012). An updated overview of clinical guidelines for chronic low back pain management in primary care. *Joint, Bone, Spine*.

[45] Cheng Z. X., Zheng Y. J., Feng Z. Y., Fang H. W., Zhang J. Y., Wang X. R. (2021). Chinese Association for the Study of Pain: expert consensus on diagnosis and treatment for lumbar disc herniation. *World Journal of Clinical Cases*.

[46] Han H., Lee D., Lee S., Jeon C., Kim T. (2015). The effects of extracorporeal shock wave therapy on pain, disability, and depression of chronic low back pain patients. *Journal of Physical Therapy Science*.

[47] Ostelo R. W., de Vet H. C. (2005). Clinically important outcomes in low back pain. *Best Practice & Research. Clinical Rheumatology*.

[48] Sun X., Ioannidis J. P., Agoritsas T., Alba A. C., Guyatt G. (2014). How to use a subgroup Analysis. *JAMA*.

[49] DIGEST Guidelines for Extracorporeal Shock Wave Therapy. https://www.shockwavetherapy.org/fileadmin/user_upload/ISMST_Guidelines.pdf.

[50] Çelik A., Altan L., Ökmen B. M. (2020). The effects of extracorporeal shock wave therapy on pain, disability and life quality of chronic low back pain patients. *Alternative Therapies in Health and Medicine*.

[51] Elgendy M. H., Mohamed M. H., Hussein H. M. (2020). Effect of extracorporeal shock wave on electromyographic activity of trunk muscles in non-specific chronic low back pain: a randomized controlled trial. *EurAsian Journal of BioSciences*.

[52] Guo X., Li L., Yan Z. (2020). Efficacy and safety of treating chronic nonspecific low back pain with radial extracorporeal shock wave therapy (rESWT), rESWT combined with celecoxib and eperisone (C+ E) or C+ E alone: a prospective, randomized trial. *medRxiv*.

[53] Taheri P., Khosrawi S., Ramezani M. (2021). Extracorporeal shock wave therapy combined with oral medication and exercise for chronic low back pain: a randomized controlled trial. *Archives of Physical Medicine and Rehabilitation*.

