# Supplementary material for: Extracorporeal Shockwave Therapy for Treating Chronic Low Back Pain: A Systematic Review and Meta-analysis of Randomized Controlled Trials
Source: Biomed Res Int. 2021 Nov 15;2021:5937250. doi: 10.1155/2021/5937250 (PMC8617566; doi:10.1155/2021/5937250)
Supplement: Supplementary 1 — Supplementary file 1: search protocol, sensitivity test, publication bias test, subgroup analysis, and meta-regression. Table A: search strategy and results of literature search. Table B: quality assessment of included studies by CBN Group risk of bias tool and Jadad score. Table C: quality of evidence assessment by GRADE. Table D: table of meta-regression results of the variables of pain intensity of 1-month follow-up. Figure A: sensitivity analysis: forest plots of pain intensity at 1-month follow-up after removing unpublished trials. Figure B: publication bias analysis: Egger's test and funnel plots of pain intensity at 1-month follow-up. [file 5937250.f1.docx]

**Supplementary file 1**: Search protocol, sensitivity test, publication bias test, subgroup analysis and meta-regression.

**Appendix 1.** Search strategies

**Appendix 2.** The quality assessment outcomes

**Appendix 3.** Sensitivity assessment

**Appendix 4.** Egger’s test and funnel plots (inspection of publication bias)

**Appendix 5.** Results of meta-regression for ESWT vs. comparatives for pain intensity at 1 month follow-up

*Overview online Tables*

- **Table A.** Search strategy and results of literature search.
- **Table B** Quality assessment of included studies by CBN group risk of bias tool and Jadad score.
- **Table C.** Quality of evidence assessment by GRADE of the included studies
- **Table D.** Table of meta-regression results of the variables of pain intensity of 1 month follow-up
- **Figure A.** Sensitivity analysis: Forrest plots of pain intensity at 1 month follow-up after removing unpublished trials
- **Figure B.** Publication bias analysis: Egger’s test and funnel plots of pain intensity at 1 month follow-up

**Appendix 1.**  **Search strategy and results of literature search**

ENGLISH DATABASE

**PubMed 67**

(SWT[All Fields] OR "shock wave"[All Fields] OR "shock waves"[All Fields] OR "extracorporeal shock wave"[All Fields] OR "shock wave therapy"[All Fields] OR "shockwave"[All Fields] OR "High-Energy Shock Waves"[Mesh] OR "Extracorporeal Shockwave Therapy"[Mesh]) AND ("back disorder"[All Fields] OR "back pain"[All Fields] OR "back ache"[All Fields] OR "backache"[All Fields] OR "lumbar injury"[All Fields] OR "lumbar pain"[All Fields] OR "lumbar ache"[All Fields] OR "lumbar injury"[All Fields] OR "lumbar facet joint pain"[All Fields] OR "lumbar myofascial pain"[All Fields] OR "lumbago"[All Fields] OR "lumbar spondylosis"[All Fields] OR "back pain"[MeSH Terms] OR "spondylosis"[MeSH Terms])

**Scopus 144**

TITLE-ABS-KEY ( swt OR esw* OR shockwave OR "shock wave" OR "extracorporeal shock wave" OR "shock wave therapy" ) AND TITLE-ABS-KEY ( "back pain" OR backache OR "lumbar pain" OR "lumbar injury" OR "lumbar facet joint pain" OR "lumbar spondylosis" OR "lumbago" OR "back disorder")

**Embase 86**

(esw*:ab,ti OR swt:ab,ti OR shockwave:ab,ti OR 'shock wave':ab,ti OR 'extracorporeal shock wave':ab,ti OR 'shock wave therapy':ab,ti) AND ('back pain':ab,ti OR backache:ab,ti OR 'lumbar pain':ab,ti OR 'lumbar injury':ab,ti OR 'lumbar facet joint pain':ab,ti OR 'lumbar spondylosis':ab,ti OR 'lumbago':ab,ti OR 'back disorder':ab,ti)

**Web of science 131**

TS=(swt OR esw* OR shockwave OR “shock wave” OR “extracorporeal shock wave” OR “shock wave therapy”) AND TS=(“back pain” OR backache OR “lumbar pain” OR “lumbar injury” OR “lumbar facet joint pain” OR “lumbar spondylosis” OR “lumbago” OR “back disorder”)

**CINAHL**: 16

S1: TI (swt OR esw* OR shockwave OR “shock wave” OR “extracorporeal shock wave” OR “shock wave therapy”) OR AB ( swt OR esw* OR shockwave OR “shock wave” OR “extracorporeal shock wave” OR “shock wave therapy” )

S2: TI (“back pain” OR backache OR “lumbar pain” OR “lumbar injury” OR “lumbar facet joint pain” OR “lumbago” OR “spondylosis”) OR AB ( “back pain” OR backache OR “lumbar pain” OR “lumbar injury” OR “lumbar facet joint pain” OR “lumbago” OR “spondylosis”)

S1 AND S2

**The Cochrane Library 43**

#1 MeSH descriptor: [Spondylosis] explode all trees

#2 MeSH descriptor: [Back Pain] explode all trees

#3 (“back pain” OR backache OR “lumbar pain” OR “lumbar injury” OR “lumbar facet joint pain” OR spondylosis OR lumbago OR “back disorder”):ti,ab,kw (Word variations have been searched)

#4 MeSH descriptor: [Extracorporeal Shockwave Therapy] explode all trees

#5 MeSH descriptor: [High-Energy Shock Waves] explode all trees

#6 (swt OR esw* OR shockwave OR “shock wave” OR “extracorporeal shock wave” OR “shock wave therapy”):ti,ab,kw (Word variations have been searched)

#7 (#1 OR #2 OR #3) AND (#4 OR #5 OR #6)

**PEDro 14**

Abstract & Title: shock*

Body part: lumbar spine, sacro-illiac joint or pelvis

Match any search term (AND)

ENGLISH DATABASE

**CNKI (China National Knowledge Infrastructure, China) -57**

Subject: shock wave AND Subject: back pain

**J-stage (Japan)-5**

Abstract: back pain AND

Abstract: shockwave or shock wave

**RISS(Research information sharing service, Korean) -16**

Basic search: shock wave back pain

TRIAL REGISTERIES

**WHO-ICTRP-11**

Basic search: shock wave AND back pain

**Clinical trials (USA) 5**

Status: All studies

Condition or disease: back pain

Other terms: shock wave

GREY LITERATURE

**Google Scholar 22**

allintitle: "back pain" "shock wave"

**Research square: 21**

Abstract: back pain

Title: (shock wave) or shockwave

**Table A. Results of literature search.**

| Data source | Results |
| --- | --- |
| *English database* | |
| Pubmed | 67 |
| Embase | 86 |
| Web of science | 131 |
| Scopus | 144 |
| CINAHL (EBSCO) | 16 |
| The Cochrane Library | 43 |
| PEDro | 14 |
| *Non-English database* | |
| CNKI (China) | 57 |
| J-Stage (Japan) | 5 |
| RISS (South Korean) | 16 |
| *Website search* | |
| Google Scholar | 22 |
| Research square (preprint) | 21 |
| *Trial registry search* | |
| WHO-ICTRP | 11 |
| Clinical trials | 5 |
| IN TOTAL | **638** |

**Appendix 2.** The quality assessment outcomes

**Table B Quality assessment of included studies by CBN group risk of bias tool and Jadad score.**

| Assessment tool | Domain | Author year  Questions | Ahmed *et al* 2018 | Çelik *et al* 2020 | Eftekharsadat  *et al* 2020 | Elgendy  *et al* 2020 | Guo *et al*  2020 | Kang *et al* 2015 | Schneider *et al*  2018 | Taheri *et al* 2021 | Walewicz *et al* 2019 | Zheng *et al* 2013 |
| --- | --- | --- | --- | --- | --- | --- | --- | --- | --- | --- | --- | --- |
| Cochrane Sources of Risk of Bias  (Yes/No/Unsure) | Selection | Was the method of randomization adequate? | Unsure | Yes | Yes | Yes | Yes | Unsure | Yes | Yes | Yes | Yes |
|  | Selection | Was the treatment allocation concealed? | Unsure | Unsure | Yes | Unsure | Yes | Unsure | No | Yes | Yes | Yes |
|  | Performance | Was the patient blinded to the intervention? | No | Yes | No | No | No | No | No | No | Yes | No |
|  | Performance | Was the care provider blinded to the intervention? | No | Yes | No | No | No | No | No | No | No | No |
|  | Detection | Was the outcome assessor blinded to the intervention? | Unsure | Yes | Yes | Unsure | Yes | Unsure | Unsure | Unsure | Yes | Unsure |
|  | Attrition | Was the drop-out rate described and acceptable? | Unsure | Yes | Yes | Unsure | Yes | Unsure | Yes | Yes | Yes | Yes |
|  | Attrition | Were all randomized participants analyzed in the group to which they were allocated? | Yes | Yes | Yes | Yes | Yes | Unsure | Yes | Yes | Yes | Yes |
|  | Reporting | Are reports of the study free of suggestion of selective outcome reporting? | Unsure | Yes | Unsure | Yes | Unsure | Unsure | Unsure | Unsure | Yes | Unsure |
|  | Selection | Were the groups similar at baseline regarding the most important prognostic indicators? | Yes | Yes | Yes | Yes | Yes | Yes | Unsure | Yes | Yes | Yes |
|  | Performance | Were cointerventions avoided or similar? | Yes | Yes | Yes | Yes | Yes | Yes | Yes | Yes | Yes | Yes |
|  | Performance | Was the compliance acceptable in all groups? | Unsure | Yes | Yes | Unsure | Yes | Unsure | Unsure | Yes | Yes | Unsure |
|  | Detection | Was the timing of the outcome assessment similar in all groups? | Yes | Yes | Yes | Yes | Yes | Yes | Yes | Yes | Yes | Yes |
|  | Other | Are other sources of potential bias unlikely? | Unsure | Yes | Yes | Unsure | Unsure | Unsure | No | Yes | Yes | Unsure |
| Jaded scale for reporting randomized controlled trials.  (Numeric Scale) | Randomization | If randomization is mentioned/appropriate? | 1 | 2 | 2 | 2 | 2 | 1 | 2 | 2 | 2 | 2 |
|  | Blinding | If blinding is mentioned/appropriate? | 0 | 2 | 1 | 0 | 2 | 0 | 0 | 1 | 2 | 0 |
|  | An account of all patients | If the fate of all patients in the trial is known or the reason of no data the is stated. | 0 | 1 | 1 | 1 | 1 | 0 | 1 | 1 | 1 | 1 |
|  | In total | | 1 | 5 | 4 | 3 | 5 | 1 | 3 | 4 | 5 | 3 |

**Table C. Quality of evidence assessment by GRADE of the included studies**

|  | | **GRADE Quality assessment** | | | | | | | **Quality** |
| --- | --- | --- | --- | --- | --- | --- | --- | --- | --- |
| No of studies | Design | | Risk of bias | Inconsistency | Indirectness | Imprecision | Publication Bias | Other considerations |  |
| **Pain at 1month** (pain scales) | | | | | | | | | |
| 10 | RCTs | | Serious^1^ | Serious^2^ | None | None^3^ | Undetected^4^ | None | ⊕⊕◯◯  LOW |
| **Pain at 3month** (pain scales) | | | | | | | | | |
| 4 | RCTs | | Serious^5^ | Serious^6^ | None | Very serious^7^ | Undetected^8^ | None | ⊕◯◯◯  VERY LOW |
| **Disability at 1month** (ODI) | | | | | | | | | |
| 5 | RCTs | | Serious^9^ | Serious^10^ | None | Serious^11^ | Undetected^12^ | None | ⊕◯◯◯  VERY LOW |
| **Disability at 3month** (ODI) | | | | | | | | | |
| 3 | RCTs | | None^13^ | None^14^ | None | Serious^15^ | Undetected^16^ | None | ⊕⊕⊕◯  MODERATE |

###### *Footnotes*

^1^ Ten studies were included. One study did not have a clear description of drop-outs (attrition bias). Six study did not have a clear description of concealment of allocation (selection bias). Eight studies did not have clear description of patient blinding, 9 studies had problems with blinding of provider (performance bias) and 6 studies did not have a clear description of blinding of outcome assessor (measurement bias). One study was industry-sponsored (other bias). Means Jadad score is 3.4. **Serious risk of bias.**

2 Heterogeneity: I² = 74%, **substantial heterogeneity**; Tau² =0.2696 (P < 0.01)

3 **Total number of participants is sufficient (n = 455)**. The 95% CI **did not include** the no effect line.

4 Publication bias was not found by funnel plot and Egger’ test.

5 Four studies were included. No studies had serious problems with drop-outs (attrition bias). One study did not have a clear description of concealment of allocation (selection bias). Two studies did not have clear description of patient blinding, 3 studies had problems with blinding of provider (performance bias) and 1 study did not have a clear description of blinding of outcome assessor (measurement bias). Means Jadad score is 4.75. **Serious risk of bias**.

6 Heterogeneity: I² = 74%, **substantial heterogeneity**; Tau² =0.2252 (P < 0.01)

7 **Total number of participants is insufficient (n = 205)**. The 95% CI **included** the no effect line.

8 Publication bias undetected.

9 Five studies were included. One study did not have a clear description of drop-outs (attrition bias). Two study did not have a clear description of concealment of allocation (selection bias). Three studies did not have clear description of patient blinding, 4 studies had problems with blinding of provider (performance bias) and 2 studies did not have a clear description of blinding of outcome assessor (measurement bias). Means Jadad score is 3.8. **Serious risk of bias.**

10 Heterogeneity: I² = 93%, **considerable heterogeneity;** Tau² =1.8277 (P < 0.01)

11 **Total number of participants is insufficient (n = 211)**. The 95% CI **did not include** the no effect line.

12 Publication bias undetected.

13 Three studies were included. No studies had serious problems with drop-outs (attrition bias). One study did not have a clear description of concealment of allocation (selection bias). One studies did not have a clear description of patient blinding, 2 studies had problems with blinding of provider (performance bias) and 1 studies did not have a clear description of blinding of outcome assessor (measurement bias). *No other issues were noticed besides the blinding and consealment.* Means Jadad score is 4.67. **Negligible risk of bias.**

14 Heterogeneity: I² = 0%, **low heterogeneity**; Tau² =0 (P =0.37)

15 **Total number of participants is insufficient (n = 114)**. The 95% CI **did not include** the no effect line.

16 Publication bias undetected.

**Appendix 3.** Sensitivity assessment

**Figure A. Forrest plots of pain intensity at 1 month follow-up after removing unpublished trials**


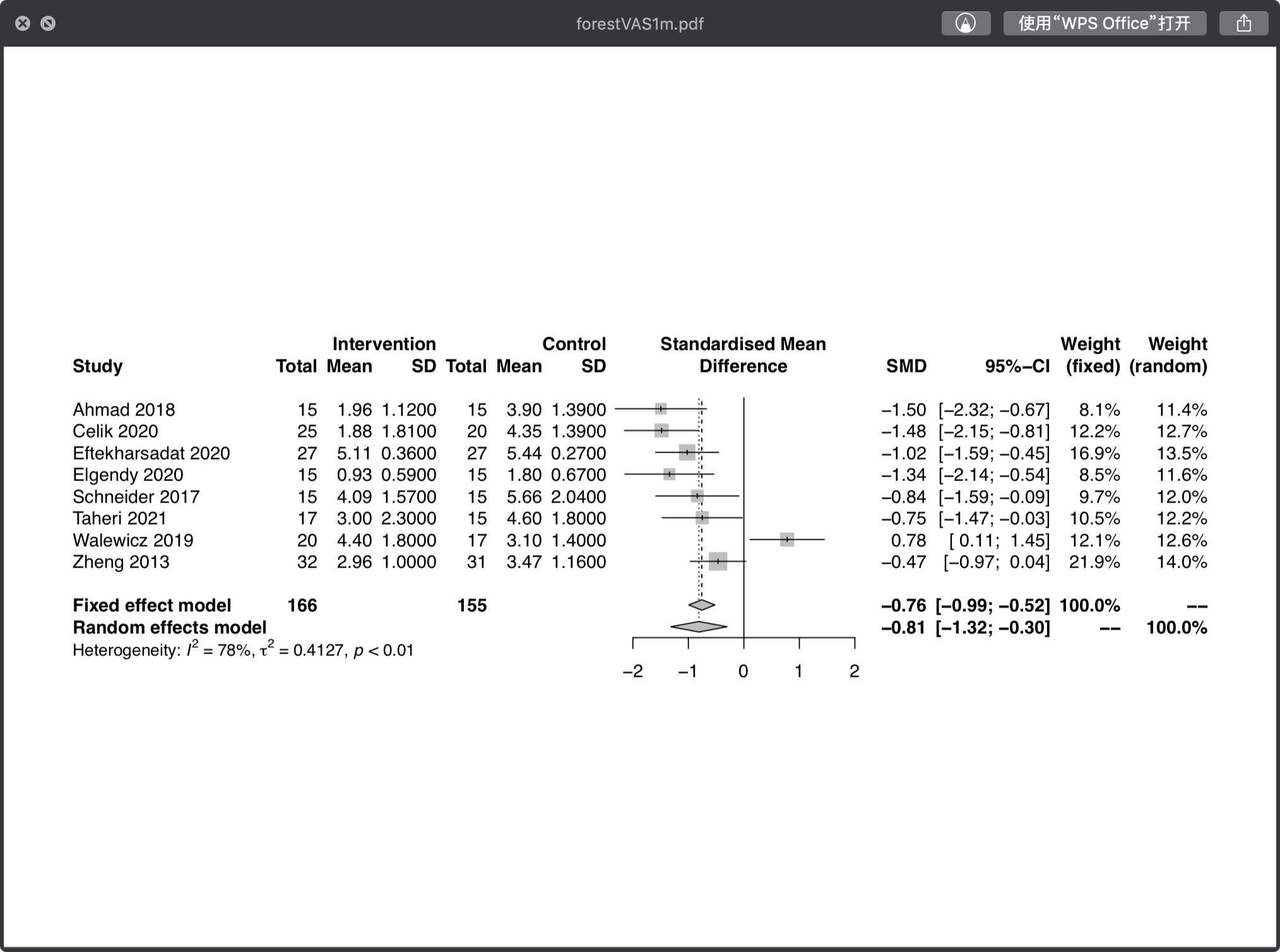


**Appendix 4.** Egger’s test and funnel plots (inspection of publication bias)

**Figure B. Egger’s test and funnel plots of pain intensity at 1 month follow-up**


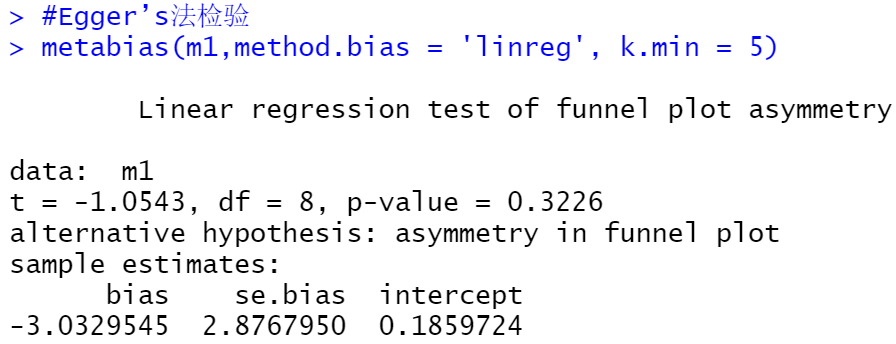

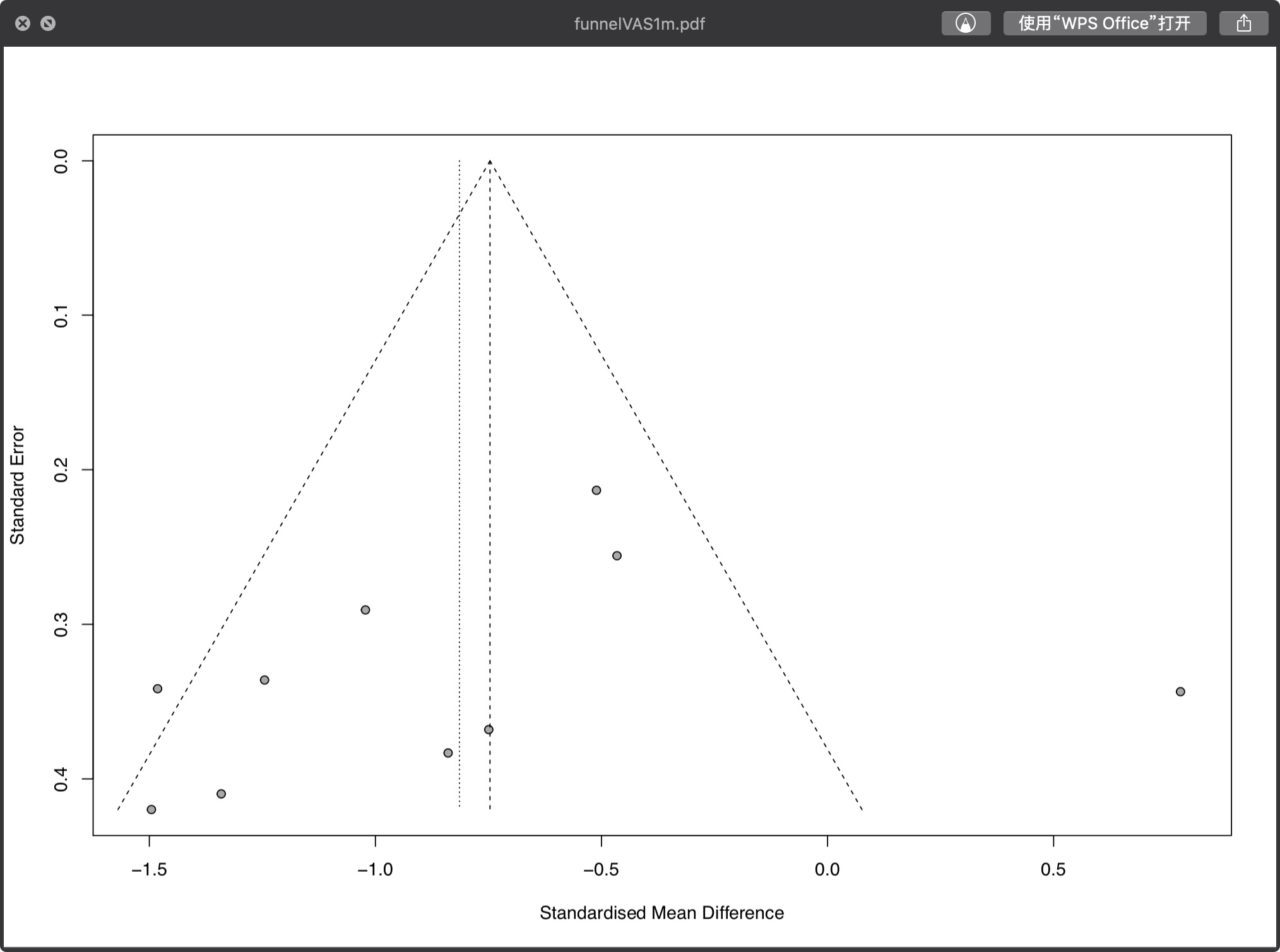


Footnote: Egger’s test (p=0.3226) and funnel plot did not show publication bias (R software package meta).

**Appendix 5.** Results of meta-regression for ESWT vs. comparatives for pain intensity at 1 month follow-up

**Table D. Table of meta-regression results of the variables of pain intensity of 1 month follow-up**

| VAS at 1 month | | | Initial model I^2^ = 74% | | |
| --- | --- | --- | --- | --- | --- |
| Variable(s) | N = 9 | B (95% CI) | P-value | I^2^ = 67% | R^2^ = 27% |
| Age |  | 0.768 (-0.056 to 1.592) | 0.068 |  |  |
| Baseline VAS |  | -0.804 (-1.653 to 0.046) | 0.064 |  |  |
| Female ratio |  | 0.232 (-0.617 to 1.080) | 0.593 |  |  |

Footnote: No significant variable was seen to contribute to the heterogeneity of estimated effects of pain intensity of 1 month follow-up.
