# Supplementary material for: Extracorporeal Shockwave Therapy for Treating Chronic Low Back Pain: A Systematic Review and Meta-analysis of Randomized Controlled Trials
Source: Biomed Res Int. 2021 Nov 15;2021:5937250. doi: 10.1155/2021/5937250 (PMC8617566; doi:10.1155/2021/5937250)
Supplement: Supplementary 2 — Supplementary file 2: data extraction form (Supplementary materials). [file 5937250.f2.pdf]

[Extracorporeal shockwave therapy for treating chronic low-back pain: a systematic review and meta-analysis of randomized controlled trials]

Data Extraction Form

Trial ID	Extractor	Year of publication
Title		
Authors		

Does this study meet all the inclusion criteria?

This is a randomized controlled trial. At least the word "random" appears somewhere in the text.
☐ yes ☐ no

The population is [ ] ☐ yes ☐ no

The intervention is [ ] ☐ yes ☐ no

The comparisons are [ ] ☐ yes ☐ no

Risk of bias assessment

Domain	Description	Review authors' judgment
**Random sequence generation**		*Selection bias (biased allocation to interventions*) due to inadequate generation of a randomised sequence<br>**Low risk/high risk/unclear**
**Allocation concealment**		*Selection bias (biased allocation to interventions)* due to inadequate concealment of allocations prior to assignment<br>**Low risk/high risk/unclear**
**Blinding of participants**<br>*Outcome:*		*Performance bias* due to knowledge of the allocated interventions by participants during the study<br>**Low risk/high risk/unclear**
**Blinding of personnel /care providers**<br>*Outcome:*		*Performance* bias due to knowledge of the allocated interventions by personnel/care providers during the study.<br>**Low risk/high risk/unclear**
**Blinding of outcomes assessors**<br>*Outcome:*		*Detection bias* due to knowledge of the allocated interventions by outcome assessors<br>**Low risk/high risk/unclear**

**Incomplete outcome data**<br>*Outcome:*		*Attrition bias* due to amount, nature or handling of incomplete outcome data<br>**Low risk/high risk/unclear**
**Selective outcome reporting**		*Reporting bias* due to selective outcome reporting<br>**Low risk/high risk/unclear**
**Group similarity at baseline**		*Selection bias* due to dissimilarity at baseline for the most important prognostic indicators<br>**Low risk/high risk/unclear**
**Co-interventions**		*Performance bias* because co-interventions were different across groups.<br>Low risk/high risk/unclear
**Compliance**		*Performance bias* due to inappropriate compliance with interventions across groups.<br>**Low risk/high risk/unclear**
**Intention-to-treat-analysis**		*Risk of bias* if all randomized patients are not reported and analyzed in the group to which they were allocated by randomization.<br>**Low risk/high risk/unclear**
**Timing of outcome assessments**		*Detection bias* if important outcomes were not measured at the same time across groups.<br>**Low risk/high risk/unclear**
**Other bias**		*Bias* due to *problems not covered elsewhere in the table.*<br>**Low risk/high risk/unclear**

Methods

(Study design including, where relevant, a clear indication of how the study differs from a standard parallel group design; duration of the study)

Participants

(Setting; relevant details of health status of participants; age; sex; country)<br><br>**Inclusion criteria:**<br><br>**Exclusion criteria:**

Intervention

(A clear list of the intervention groups included in the study)

Experiment group:

Control group:

Additional information requested

Notes

Outcomes

	Outcome Measures (Continuous)	Total participants N =					
		Intervention group n =			Control group n =		
		total	mean	SD	total	mean	SD
	**Primary (pain intensity):**						
1	VAS						
2	NRS						
3	Others						
	**Secondary (disability score):**						
4	ODI						
5	EQ-5D						
6	Others						

	Outcome Measures (Dichotomous)	Total participants N =			
		Intervention group n =		Control group n =	
		events	total	events	total
	Primary (adverse events):				
1					
